# Novel Molecular Markers and Immune-Related Candidate Genes for Blackleg Resistance in Rapeseed: A Genome-Wide Analysis

**DOI:** 10.3390/ijms27062567

**Published:** 2026-03-11

**Authors:** Ewa Starosta, Tomasz Jamruszka, Justyna Szwarc, Jan Bocianowski, Magdalena Grynia, Janetta Niemann

**Affiliations:** 1 Department of Genetics and Plant Breeding, Poznań University of Life Sciences, Dojazd 11, 60-632 Poznań, Poland; ewa.starosta@up.poznan.pl (E.S.); tomasz.jamruszka@up.poznan.pl (T.J.); justyna.szwarc@up.poznan.pl (J.S.); 2 Department of Mathematical and Statistical Methods, Poznań University of Life Sciences, Wojska Polskiego 28, 60-627 Poznań, Poland; jan.bocianowski@up.poznan.pl; 3 IHAR Group, Borowo Department, Strzelce Plant Breeding Ltd., Borowo 35, 64-020 Czempiń, Poland; m_grynia@hr-strzelce.pl

**Keywords:** *Brassica napus*, blackleg resistance, DArTseq, GWAS, marker-assisted selection, SNP, SilicoDArT, next-generation sequencing

## Abstract

Rapeseed (*Brassica napus* L.) faces escalating threats from abiotic and biotic stresses, notably blackleg caused by *Leptosphaeria maculans*. Due to limited chemical control efficacy and stringent GMO regulations, marker-assisted selection (MAS) leveraging natural genetic variation has become an indispensable strategy for crop improvement. This study identified novel molecular markers for blackleg resistance by integrating genome-wide association study (GWAS) results with high-throughput genotyping by Diversity Arrays Technology sequencing. Phenotypic screening across the population demonstrated a wide spectrum of disease severity (scores 0–6), confirming the segregation of key resistance genes. The DArTseq platform identified nearly 104,000 markers, comprising 61% SilicoDArTs and 39% SNPs. Among the 33 most significant markers associated with resistance (*p* < 0.01), 76% were SilicoDArTs. Transcriptomic data further validated these findings, revealing 13 marker-linked genes expressed during infection, seven of which exhibited significant differential expression. Comprehensive functional annotation of *Arabidopsis thaliana* orthologs associated these genes with diverse cellular and plant-wide processes, particularly during stress responses. Collectively, these findings emphasize the complex polygenic nature of blackleg resistance and provide robust genomic tools for the accelerated breeding of resilient *B. napus* cultivars.

## 1. Introduction

Rapeseed (*Brassica napus* L.) is considered one of the most significant cultivated plants within the *Brassica* genus [1]. It belongs to the group of three allotetraploid species formed through hybridization among the core “U’s triangle” species: *B. nigra* (2n = 2x = 16 = BB), *B. rapa* (2n = 2x = 20 = AA), and *B. oleracea* (2n = 2x = 18 = CC) [2]. Moreover, rapeseed is regarded as one of the most important oilseed crops. Contemporary cultivars yield substantial amounts of oil characterized by high nutritional quality. In addition to its use for oil production, rapeseed is also valued as a source of animal feed and as a raw material for biofuel generation [3,4,5]. Initially, rapeseed oil was unsuitable for human consumption because of its high levels of erucic acid and glucosinolates. However, breeding efforts resulted in the development of “double zero” (“00”) cultivars, characterized by very low concentrations of these compounds [6].

Intensive inbreeding in *B. napus* aimed at improving oil content and quality has significantly reduced its genetic diversity. Consequently, the species has become increasingly susceptible to a wide range of biotic stress factors, including fungi, viruses, and insect pests [7]. Several diseases have become major constraints in rapeseed production. Among them, blackleg is considered one of the most economically important. The disease is caused by a fungal complex of *Leptosphaeria maculans* and *Leptosphaeria biglobosa.* The first symptoms of infection appear as oval, gray lesions with black pycnidia spots on cotyledons and leaves. As the disease progresses, similar lesions develop on stems and siliques. In the most advanced stages of infection, a characteristic blackening of the basal stem is observed, which is associated with significant yield losses [8].

Global yield losses in rapeseed caused by this disease are estimated at 10–15%, though in extreme cases, they may reach up to 90%. Various control strategies have been developed and applied in rapeseed cultivation, including agricultural practices such as tillage and crop rotation, as well as biological and chemical protection methods, involving antagonistic microorganisms and fungicide applications [9].

The use of resistant rapeseed varieties remains the most cost-effective and environmentally sustainable strategy for managing blackleg disease. Although numerous studies have investigated the genetic basis of resistance in rapeseed, current knowledge only scratches the surface. The molecular mechanisms underlying plant immunity are highly complex and involve a wide array of genes, ranging from those with minor effects to those with major effects [3,7]. In agriculture, rapeseed varieties carrying well-characterized resistance genes are widely cultivated as a means of protecting crops against *L. maculans*, the causal agent of blackleg disease. To date, researchers have identified 22 major resistance (*R*) genes in *B. napus* and related species [10]. These genes interact in a classic gene-for-gene manner with the corresponding avirulence (*Avr*) genes of the pathogen, triggering defense responses in the host [11]. However, the intensive use of single resistant cultivars in monoculture, combined with limited rotation of different *R* genes, has created strong selective pressure on pathogen populations. As a result, new virulent isolates capable of overcoming specific resistance genes have rapidly emerged, leading to a breakdown of *R*-gene-mediated resistance and a reduction in the long-term effectiveness of genetic resistance in rapeseed breeding programs [10,12].

Therefore, it is crucial to intensify ongoing research aimed at identifying new major resistance (*R*) genes as well as quantitative trait loci (QTLs) associated with partial resistance to *L. maculans* [13]. Unlike single *R* genes, which often lose their effectiveness due to pathogen adaptation, QTLs generally confer a broader and more durable form of resistance by reducing the overall disease severity rather than providing complete immunity. When both types of resistance are strategically combined—major *R* genes ensuring strong initial protection and QTLs contributing to long-term stability—the resulting cultivars display the highest and most sustainable level of defense against blackleg disease [14]. Such an integrated approach represents a key strategy for maintaining effective crop protection and ensuring stable yields in rapeseed production.

Many breeding programs and stations emphasize the urgent need for reliable and efficient molecular markers to enable rapid and straightforward screening of *B. napus* germplasm for blackleg resistance. Such tools would greatly facilitate the development of elite cultivars that combine strong disease resistance (conferring both *R*-genes and QTLs) with high economic and agronomic value.

Marker-assisted selection (MAS) has become one of the main methods of elite germplasm selection. Nevertheless, the highly complex nature of the *B. napus* genome poses a significant challenge for blackleg resistance research. As an amphidiploid species, *B. napus* combines two parental genomes (*B. rapa* and *B. oleracea*), resulting in extensive genome size, polyploidy, and a high degree of gene redundancy. This complexity complicates the precise identification and mapping of resistance loci, as well as the development of reliable molecular markers tightly linked to blackleg resistance. Consequently, distinguishing functional resistance genes from closely related paralogs and pinpointing their exact chromosomal positions requires advanced genomic tools, large-scale mapping populations, and integrative approaches combining genomics, transcriptomics, and bioinformatics.

Diversity Arrays Technology sequencing (DArTseq) is a cost-effective genotyping-by-sequencing platform that reduces genome complexity using restriction enzymes, followed by adapter ligation, amplification, and high-throughput sequencing. This approach enables the detection of thousands of SNPs and presence–absence variants without requiring a reference genome. Consequently, DArTseq is particularly well suited for genetic diversity analysis, linkage mapping and marker-assisted breeding in crops with complex genomes.

Doubled haploid (DH) rapeseed lines provide an optimal population structure for such analyses due to their complete homozygosity and genetic stability. Their fixed genotypes allow unambiguous evaluation of associations between molecular markers and blackleg resistance. Moreover, the use of DH lines reduces the required population size, which is necessary for reliable detection of qualitative and quantitative resistance loci while maintaining high power for detecting both qualitative and quantitative resistance loci. The integration of DH populations with DArTseq facilitates precise marker–trait association analysis. The genetic uniformity of DH lines ensures clear and reproducible marker signals, thus increasing the reliability of identified markers and supporting their effective application in MAS and resistance gene pyramiding.

In recent decades, numerous molecular markers linked to blackleg resistance have been reported using a wide range of approaches, including biparental linkage mapping, candidate gene analysis, association mapping, and high-throughput genotyping platforms. These studies have led to identification of markers associated with both major resistance (*R*) genes and quantitative trait loci (QTLs), many of which are located within or near genes involved in plant defense pathways [14,15]. While these findings have substantially advanced the understanding of the genetic basis of resistance, their practical implementation in breeding programs has been comparatively limited. Many reported markers exhibit population specificity, reduced effect sizes in diverse genetic backgrounds, or insufficient validation across environments and germplasm panels [16]. Therefore, only a relatively small proportion of identified markers have proven robust and transferable enough for routine application in MAS.

This study aims to (i) identify novel molecular markers associated with blackleg resistance in rapeseed DH lines by employing next-generation sequencing in combination with physical mapping and (ii) conduct a preliminary validation of their association with disease resistance through in silico functional annotation of candidate genomic regions. The primary objective is to identify SNP and SilicoDArT markers linked to blackleg resistance. By integrating DArTseq genotyping with precise phenotyping of DH lines, we aim to map new resistance loci within the *B. napus* genome. Furthermore, with functional inference, we aim to provide biologically informed interpretation of marker–trait associations and support their prospective application in resistance breeding programs. These findings will provide robust tools for MAS, enabling the efficient development of rapeseed cultivars with enhanced and durable resistance to *L. maculans*.

## 2. Results

### 2.1. Phenotyping

**Figure 1:**
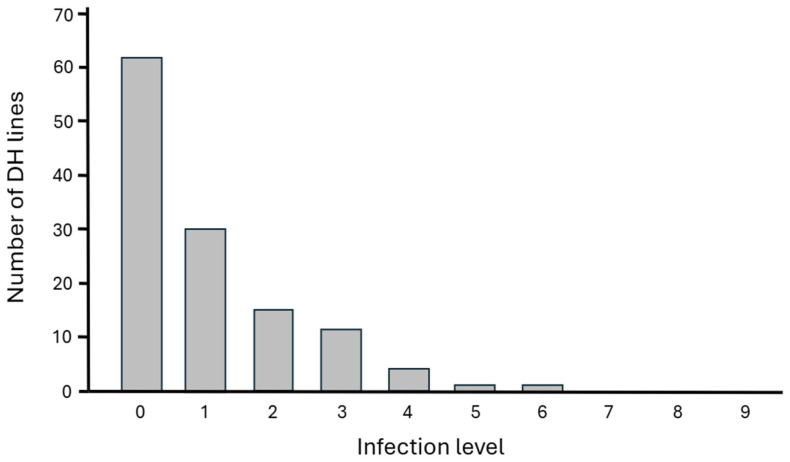
Blackleg infection level in 125 analyzed *Brassica napus* doubled haploid (DH) lines. Infection scored on a 0 (no damage) to 9 (completely damaged plant) scale.

The level of resistance of the 125 analyzed DH lines was assessed using a 10-point severity scale, where extreme values of 0 and 9 indicate no visible disease damage and a completely damaged plant, respectively. Varying infection levels, ranging between 0 and 6, were observed with an average of 0.96 ± 1.27. Most plants exhibited very low or no infection. None of them were severely affected, i.e., at a level of 7–9 (Figure 1).

### 2.2. Genotyping

**Figure 2:**
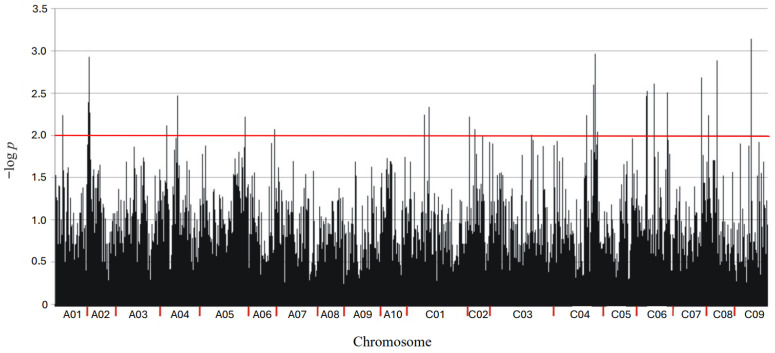
Manhattan plot for polymorphic markers associated with resistance of *B. napus* to blackleg. The horizontal red line indicates the genome-wide significance threshold (−log_10_(*p*) = 2). Markers above this line are considered significantly associated with the trait.

Through DArTseq, a total of 103,898 molecular markers (60,067 SilicoDArT and 43,831 SNP) were obtained. Based on a significance threshold of MAF > 0.25 and less than 10% missing observations as well as chromosomal location, 14,759 markers (10,314 SilicoDArTs and 4445 SNP) were selected and used for association mapping (Figure 2).

**Figure 3:**
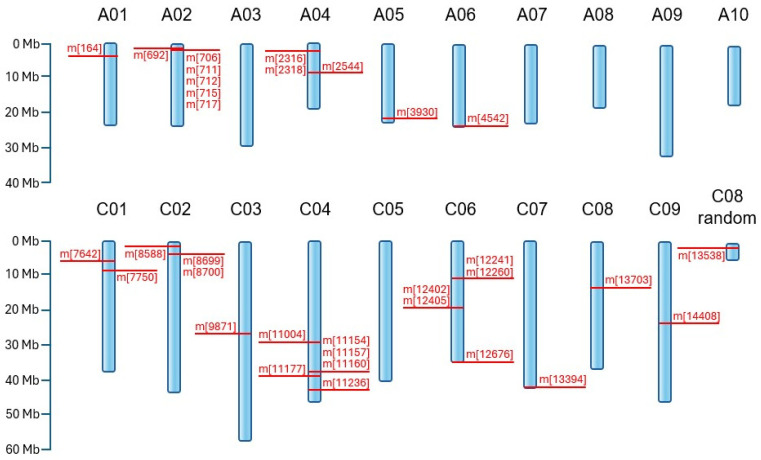
A chromosomal distribution of markers linked to blackleg resistance in *B. napus* at *p* < 0.01. The blue structures represent chromosomes, labeled with their respective numbers at the top. Markers and their specific genomic coordinates are highlighted in red, with positions expressed in megabases (Mb) according to the provided scale.

A highly significant association (*p* < 0.01) between 33 markers (25 SilicoDArT and 8 SNP) from this group and plant resistance to *Leptosphaeria* spp. was observed (Table 1). Chromosomes A02 and C04 contained the highest number of identified markers (6 each), while no marker was found on chromosomes A07 to A10 and C05 (Table 1 and Figure 3).

**Figure 4:**
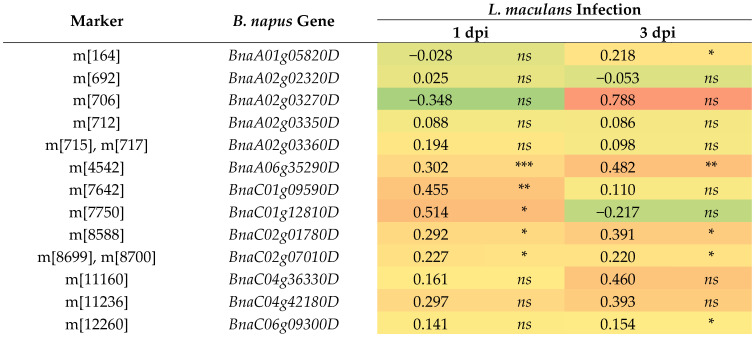
A heatmap showing changes in the expression levels of fifteen *B. napus* genes that harbor selected markers caused by *Leptosphaeria maculans* 1 and 3 days post-inoculation (dpi). The heatmap values depict log2-fold changes in transcriptomic data acquired from Becker et al. (2019) [15]. Red and orange indicate upregulated gene expression; green represents downregulated gene expression; yellow denotes stable expression levels. Significant differences from the mock-treated plants were determined using two-tailed Student’s *t*-test. * *p* < 0.05; ** *p* < 0.01; *** *p* < 0.001; *ns*, not significant.

Out of all the markers identified, 21 markers (16 SilicoDArT and 5 SNP) were intragenic. Nearly all of these markers corresponded to only one gene. The exceptions were the SilicoDArT marker m[715] and the SNP marker m[717], which were found within a subsequent intron and exon of *BnaA02g03360D*, respectively. Additionally, two SNP markers, m[8699] and m[8700], were localized within an adjacent exon and intron of another gene, *BnaC02g07010D* (Table 2). Among the 19 genes harboring markers, 15 were identified in the transcriptomic data from Becker et al. (2019) [17], which assessed gene expression changes in response to *L. maculans* infection. Notably, eight of these genes showed a statistically significant reaction to the pathogen. Additionally, a few others (*BnaA02g03270D*, *BnaC04g36330D*, and *BnaC04g42180D*) exhibited a noticeable though not statistically significant reaction (Figure 4).

## 3. Discussion

Rapeseed (*B. napus* L.) is a globally significant crop due to its high-quality oil and protein content [3]. Unfortunately, the expansion of the rapeseed cultivation area leads to an increased risk of production disturbances caused by abiotic and biotic stresses, including pathogenic fungi [7,18]. Given the often-limited scope and low efficacy of agronomical interventions, as well as environmental concerns associated with the utilization of chemical plant protection products, solutions based on genetically mediated plant resistance are being increasingly explored [19,20]. One of the methods for the genetic improvement of economically important plant varieties, including rapeseed, is MAS. It remains a key strategy for *B. napus* improvement, leveraging natural genetic variation to bypass the regulatory complexities of genome editing while maintaining high breeding efficiency [21]. The efficacy of MAS increases with the advent of newer high-throughput marker detection technologies. Thus, this study describes the procedure and results of the identification of novel molecular markers for blackleg resistance in rapeseed by integrating Genome-Wide Association Study (GWAS) results from phenotypic screening and genotyping via DArTseq.

The use of DH lines in this study eliminated the need to distinguish signals derived from two different alleles (heterozygosity), which can be problematic in NGS analysis. This increases the reliability of read mapping and genotyping, because the fully homozygous nature of DH lines enhances the precision of variant calling, facilitating the subsequent and accurate identification of linked genes and QTLs [22].

The low mean disease severity observed in the analyzed plants could be attributed to a few factors: either the population possesses high innate resistance, or there was potentially low inoculum pressure of the pathogen in the field. Fortunately, the wide range of symptoms that were also observed is crucial for genetic research. The disease severity across the analyzed plant population exhibited high variability, ranging from a score of 0 to 6. This spectrum covers everything from completely healthy plants with no visible disease symptoms (score 0) up to those displaying severe symptoms—specifically, discoloration covers 51% to 75% of the main stem and is accompanied by numerous pycnidia (score 6). The high variability observed confirms the segregation of genes responsible for resistance, which makes the population suitable for genetic mapping, e.g., QTL analysis [23] (Figure 1).

The effectiveness of the DArTseq platform in dissecting complex traits is well-documented across various *B. napus* studies [24,25,26]. Increasingly, this technique is being utilized to study this species in economically relevant contexts. Examples of such work include studies on pod shatter resistance [27,28], aluminum stress tolerance [29], Mn^2+^ tolerance [30], and blackleg resistance. The latter has been a particularly active area of research, with studies using DArTseq markers to identify and map *R* genes [31] and GWAS [32].

By employing DArTseq, a level of genomic resolution was achieved that far exceeds the capabilities of traditional marker systems like RFLP or RAPD. This high-throughput platform enabled the simultaneous interrogation of tens of thousands of loci, providing the dense marker coverage necessary to dissect the complex, polygenic nature of blackleg resistance in *B. napus*. In addition to high efficiency, the integration of sequence-specific data allowed for the direct anchoring of the findings to the reference genome—a critical step for precise marker localization that could be largely infeasible with earlier molecular tools [33].

The DArTseq technology generates molecular markers, which are primarily classified into two types: SilicoDArTs, based on presence or absence in the pool of analyzed genotypes, and SNPs present in the representation. Furthermore, it is also possible to extract Copy Number Variation (CNV) polymorphism information (https://www.diversityarrays.com/) [34]. In this study, over 100 thousand molecular markers were identified, of which 58% were SilicoDArT-type and 42% were SNP-type. The ratios among the 33 most significant markers associated with blackleg resistance with the highest significance (*p* < 0.01) were 76% and 24%, respectively (Table 1). However, the actual content of markers containing single or multiple SNPs in their structure might be greater. This is due to the lack of precise specification regarding exactly what polymorphism contributes to SilicoDArT, as it can contain SNP or an indel rather than exclusively a fragment presence/absence not linked to a point mutation. A case supporting this possibility, which warrants further investigation, is the pair of identified markers m[715] and m[717]. The SNP for the first marker, m[715], is located in the 9th intron of the gene *BnaA02g03360D*, while the second marker, m[717], is a SilicoDArT marker whose sequence covers a fragment of the same gene spanning from the 9th exon to the 9th intron. This positional overlap suggests that the two different marker types may linked to the same polymorphism [34] (Table 2).

In this study, the higher frequency of SilicoDArT markers compared to SNPs stems from both the DArTseq methodology and the inherent nature of the DH lines. Because DH lines are fully homozygous, they lack heterozygous loci; consequently, genetic variation is expressed exclusively through fixed allelic differences.

SilicoDArT markers, which operate on a presence/absence basis, are particularly effective here as they detect not only point mutations at restriction sites but also broader structural changes like insertions and deletions. In contrast, while SNP calling in these lines is precise, it is technically more demanding, requiring high read depth to ensure reliability. Therefore, the binary nature of SilicoDArT markers provides a more comprehensive and robust dataset for high-density genetic mapping in homozygous populations.

The molecular markers identified in this study were distributed across multiple chromosomes of both the A and C subgenomes (A01, A02, A04, A05, A06, C01, C02, C03, C04, C06, C07, and C09), highlighting the polygenic and genome-wide nature of blackleg resistance in *B. napus*. Notably, none of the detected markers were located within the well-characterized A07 resistance cluster, which harbors several major *R*-genes (*Rlm1*, *Rlm3*, *Rlm4*, *Rlm7*, and *Rlm9*), nor within the A10 region containing *Rlm2* and *LepR*-associated loci [35,36,37]. Our results revealed resistance-associated loci distributed across both subgenomes, including multiple C-genome chromosomes (C01, C02, C03, C04, C06, C07, and C09). Previous reports indicate that C-genome loci are frequently associated with moderate, additive effects contributing to partial but potentially more durable resistance [38]. The broad chromosomal distribution observed here suggests a complex genetic architecture involving multiple loci with cumulative effects rather than reliance on single major *R*-genes.

Among the 19 characterized genes harboring markers, 13 were found in the transcriptomic data from Becker et al. (2019) [17]. Seven of these exhibited a statistically significant change in expression 1 and/or 3 days following infection with *L. maculans*. Additionally, some genes displayed prominent, though not statistically significant, changes in expression. The reason for the lack of statistical significance, or indeed, the complete absence of a reaction to the tested pathogen, might be that the gene’s activity is primarily restricted to tissue other than the one analyzed (i.e., not the cotyledons), or that the timing of the reaction to the tested stimulus might be different (i.e., earlier or later than the 1- and 3-day time points) [39]. Furthermore, the presence of markers in intergenic regions may indicate that markers are located within regulatory elements (like enhancers or promoters) that control the expression of adjacent or distant genes, thus influencing the plant’s reaction to the pathogen without being a part of the coding sequence itself [40] (Figure 4).

Functional annotation of identified loci suggests that blackleg resistance in *B. napus* is a multifaceted process involving several key pathways. Stress signaling and transcriptional regulation are pivotal, evidenced by the presence of OBF5 (systemic acquired resistance activation) [41], HSF3 (stress signal transduction) [42], XPO1A (antioxidant defense) [43], and FBS2 (protein degradation) [44,45]. These findings point toward a comprehensive and coordinated defense response (Figure 4 and Table 3).

Additionally, structural and metabolic adaptations appear crucial, with genes linked to cell wall integrity (*SFH19*) [46], cytoskeleton organization (*At5g14790*), and sugar transport (*At5g17010*, *VGT2*) [47,48] supporting cellular barriers. Finally, the involvement of fundamental maintenance genes, including *REIL1* (ribosome remodeling) [49,50], *INH3* (dephosphorylation) [51], and *At1g51980* (electron transport) [52], highlights the high metabolic cost of this defense. Together, these diverse pathways—from DNA damage response (*PP4R2L*) [53] to salt-stress factors (*GT-4*) [54]—indicate a broad-spectrum physiological adaptation that enhances resistance to *L. maculans* (Figure 4 and Table 3).

The clustering of markers around candidate genes suggests that structural changes within these markers may drive the differences in resistance between the DH lines. These modifications likely alter gene expression or lead to the gain or loss of specific defense receptors. Within a theoretical defense network, these genes may act as key nodes, where structural variations determine how quickly and strongly the plant triggers a resistance response.

While the biological roles of these candidate genes are well-documented in *A. thaliana*, their specific functions in *B. napus* during *L. maculans* infection remain to be experimentally confirmed. These inferred roles provide a strong basis for future functional validation studies, such as virus-induced gene silencing (VIGS) or CRISPR/Cas9-mediated mutagenesis, to definitively link these loci with blackleg resistance.

The ongoing research into blackleg resistance in *B. napus* exemplifies the crucial intersection of advanced genotyping technologies and effective breeding strategies. By employing innovative methods such as DArTseq and GWAS, the resilience of *Brassica* crops against key diseases is progressively enhanced, thereby safeguarding yield and contributing to food security. This study successfully identified a substantial repertoire of SilicoDArT and SNP markers, many of which are situated within or adjacent to genes associated with critical stress response pathways. The functional diversity of these genes and their respective proteins—ranging from transcriptional regulation (HSF3 and OBF5) to structural integrity (XPO1A and SFH19)—underscores the multifaceted nature of the plant’s defense mechanisms. These findings not only expand our understanding of the molecular basis of *L. maculans* resistance but also provide a suite of validated molecular markers for MAS. Consequently, these results offer a practical pathway for breeding resilient rapeseed varieties, ensuring stable yields in the face of escalating biotic pressures.

## 4. Materials and Methods

### 4.1. Plant Material

The plant material consisted of 125 DH rapeseed lines (*B. napus* L.) from Strzelce Plant Breeding Ltd., Borowo Department (Borowo, Poland), IHAR Group, developed via intraspecific crosses of registered varieties with known blackleg resistance to ensure a diverse disease response.

### 4.2. Field Assessment

Field evaluations were carried out to assess disease severity and determine the blackleg resistance of all analyzed DH lines. The experiments took place at the test fields located in Borowo, Wielkopolska voivodeship, Poland (Strzelce Plant Breeding Ltd., IHAR Group). The study was carried out using a randomized block design and included three replications. The size of a single testing plot was 10 m^2^ with a sowing density of 60 seeds/m^2^. The agricultural practices were optimal for local conditions. Resistance observations were performed on mature plants during the BBCH 70–89 phase. The degree of resistance to blackleg was assessed by rating disease symptoms based on the 0–9 scale developed by Jędryczka (2006) [55]. On this scale, a score of 0 signifies the absence of visible disease symptoms, while a score of 9 indicates a completely damaged plant with numerous pycnidia present on the stems and leaves.

### 4.3. DNA Extraction

Genomic DNA was isolated from 7-day seedlings using the Genomic Mini AX Plant kit (A&A Biotechnology, Gdańsk, Poland), according to the supplied protocol. Samples exhibiting 260/280 and 260/230 ratios around 1.8 and 2.0, respectively, and concentrations above 100 ng/uL were diluted to a final concentration of 100 ng/uL.

### 4.4. Genotyping

The genomic DNA isolated from the analyzed DH lines was subjected to DArTseq analysis by Diversity Arrays Technology (University of Canberra, Canberra, Australia). The procedure includes (i) reduction in genome complexity through digestion with appropriate species-specific restriction enzymes, (ii) ligation of resulting fragments with adaptors, (iii) amplification using polymerase chain reaction (PCR), (iv) preparation of gene pool representation by combining equimolar quantities of amplification products from each genotype, and (v) sequencing with Illumina platform (Illumina, San Diego, CA, USA).

Following initial quality assessment via the DArT analytical pipelines (Diversity Arrays Technology, Canberra, Australia), the markers were subjected to stringent selection criteria for the association study. Only those meeting the following requirements were retained: one marker per 69 nt sequence, a minor allele frequency (MAF) > 0.25, and a missing observations fraction < 10%.

### 4.5. Physical Mapping and Gene Annotation

Marker positions were physically mapped against the Darmor-bzh v4.1 reference genome. Subsequently, the resulting metadata, specifically, the presence/absence and chromosomal localization of the identified SilicoDArTs and SNP markers, were subjected to statistical analysis. Moreover, the distances between the markers and the nearest genes were measured using the JBrowse tool available on the *B. napus* multi-omics information resource (BnIR, https://yanglab.hzau.edu.cn/BnIR, accessed on 15 January 2026). The functional role of the selected genes regarding their potential contribution to *B. napus* defense mechanisms against *L. maculans* was assessed through re-analysis of pre-existing transcriptomic data from Becker et al. (2019) [17].

### 4.6. Statistical Analysis

Data were further subjected to analysis of variance in a model with random effects of DH lines. Using the method based on the mixed linear model with a population structure calculated by eigenanalysis and modeled by random effects, association mapping was carried out based on SilicoDArT and SNP data and average trait values [56]. The results were analyzed and are shown in GenStat 23.1 [57] using the QSASSOCIATION method, which uses data from a single-environment experiment to conduct a mixed-model marker–trait association analysis (sometimes called linkage disequilibrium mapping). Some kind of genetic relatedness control is required in association mapping investigations to prevent false positives. The RELATIONSHIPMODEL = eigenanalysis option was used to specify the model, which uses the most significant principal components from the molecular marker matrix to infer the underlying genetic substructure in the population [58]. The scores of the significant axes are used as covariables in the mixed model, which is essentially an approximation to the structuring of the genetic variance covariance matrix by a coefficient of coancestry matrix (kinship matrix). A false discovery rate (FDR) adjusted at 0.05 was initially used to determine the *p*-value threshold for declaring significant marker–trait associations. However, due to the high stringency of FDR-adjusted *p*-values and the potential risk of type II error, the criterion of selecting the *p*-values obtained within the bottom 0.1 percentile of the distribution was utilized. Thus, a threshold of *p* < 0.001 was used to declare significant marker–trait associations. The Benjamini–Hochberg technique was used to adjust *p*-values for multiple testing in order to determine the significance of the association between blackleg and SilicoDArT and SNP markers.

## Figures and Tables

**Table 1:** SilicoDArT and SNP molecular markers significantly associated with resistance of *Brassica napus* to blackleg at *p* < 0.01.

Marker	Marker Type	DNA Strand	Chromosome	Marker Position on Chromosome (bp)	Marker Sequence *
m[164]	SilicoDArT	Minus	A01	2,712,099–2,712,168	TGCAGTAGAGACACATGAAGTCTCTCTTGACCTGAATGATGGATCCATGGTATAAAGAGTAAATAGGAG
m[692]	SilicoDArT	Plus	A02	1,028,068–1,028,137	TGCAGACATCTTCAGCGCGTATAACAACGATATAACAGATCTGTTGGATTCTCTGGTAATTCTTTTCAT
m[706]	SilicoDArT	Minus	A02	1,455,169–1,455,238	TGCAGCTGGTGTTCCGTTGCTTGTTGCTGCTGCCTCAGCCTCACAAGCTCTTGCATAAGAACATTCTTG
m[711]	SilicoDArT	Plus	A02	1,481,265–1,481,326	TGCAGGTTGTACTGCGAGATCATTCCACACGCTGCGATGCGGCCGTGTGGTCTCATGTTAC
m[712]	SNP	Minus	A02	1,484,605–1,484,674	TGCAGGTGATGCAACAAGGGTCTCAATTCTACTTGGTCTATTGAAGGTATTCAGTTTTCTTGACCTGTT
m[715]	SNP	Plus	A02	1,496,129–1,496,198	TGCAGGTATATTAGTTATCTTCTTGTATTATCATCTTTTTGCTCGTTGACATTCCGACTCTTCTAGTTT
m[717]	SilicoDArT	Plus	A02	1,496,129–1,496,198	TGCAGGTATATTAGTTATCTTCTTGTATTATCATCTTTTTGCTCGTTGACATTCCGACTCTTCTAGTCA
m[2316]	SilicoDArT	Plus	A04	2,485,759–2,485,804	TGCAGTACATGCAGCCACTTTCGTCATCAGTTTTTTTTTTTTTAC
m[2318]	SilicoDArT	Plus	A04	2,532,015–2,532,052	TGCAGACATATTTGGATACTAACCGTGGTCCGGTTAC
m[2544]	SNP	Minus	A04	9,341,987–9,342,056	TGCAGTTGACCTTGAAATCCGGGTGGCCACACTCTTTTCTCTCAGGTATCCAAAAGGGATAACTGAGAT
m[3930]	SilicoDArT	Minus	A05	21,784,960–21,785,029	TGCAGATTGCAGAAAGCATCTATGCTTTCTGATTTTCAGACACTCCCAAAACCCCAAAAGAAAAAAGTG
m[4542]	SilicoDArT	Minus	A06	23,251,856–23,251,925	TGCAGCCAGACAGAGAGAGTTCCCCAGAGAGAAGTAAAAAATCTCCAAAGATCGACTCTCTTTTTTCTG
m[7642]	SilicoDArT	Plus	C01	5,611,916–5,611,847	TGCAGAGCACAAAGAACCAGCTTCAGTCAGTTTCAGTGATCACAGGGACGAAAACTAATATTACGTATT
m[7750]	SilicoDArT	Plus	C01	8,202,631–8,202,700	TGCAGAACCAAAGCTCACCGATCAAATGTAGATAATGAATCATCAGAACACAGAGAAAAAAAAAAAAGA
m[8588]	SilicoDArT	Minus	C02	772,187–772,256	TGCAGTACGATGGTGGGCATGTGGGTGAATCTAGCGACCGTTCAAAGGAAAAAATGGATCAAAAGGTAA
m[8699]	SNP	Plus	C02	3,734,120–3,734,154	TGCAGAGTAAATGGAGGACCTTCGTCGAAATTAC
m[8700]	SNP	Minus	C02	3,734,054–3,734,123	TGCAGAGACTCTGTGAGGTAAGTAGATGTGGTTGCTCATCGTGATTTACTTCAGTGTAGGAGATATCAT
m[9871]	SilicoDArT	Minus	C03	27,022,233–27,022,281	TGCAGGATCAATGGGACTGTTTGGGAACTACCAAGTGAGTCTTTTTAC
m[11004]	SilicoDArT	Minus	C04	28,776,225–28,776,294	TGCAGAAGAAGTATTGGCACATAGTGGATATCCCTCTGGTGGTGAATGAATGGTCTCCGGAGACTGCAA
m[11154]	SilicoDArT	Minus	C04	37,643,829–37,643,898	TGCAGCTATACCCCGCAGCAGAATGAGGTCTCAGAAAGGATGAACAGAACCATCATGGATAAAGTGAGA
m[11157]	SilicoDArT	Plus	C04	37,746,393–37,746,462	TGCAGACCAACTCCATAGGATCATTGATAAAATGATGAGCAACAGAATAAGCTCTTTGAAGTATACCGG
m[11160]	SilicoDArT	Plus	C04	37,865,298–37,865,363	TGCAGAAAGCTAGAAGATAGACTCCTTTTTTGTGTGAATATGGTCAGAGACTGATAGACTTTTAC
m[11177]	SilicoDArT	Minus	C04	39,051,898–39,051,967	TGCAGAGATAGGAGACGGTTGCGGAAATGTTTGTCCAGCTCTCGAGTTAGGTGTTCTGCTGATGTTGGT
m[11236]	SilicoDArT	Plus	C04	42,817,467–42,817,536	TGCAGCGGTGGGAGACAAAAAAAGAAAGAAGGATCAATTCCCAAAGACTTGTTTCTTTGTTGTAAAGCC
m[12241]	SilicoDArT	Plus	C06	10,711,436–10,711,490	TGCAGCTAAACTCAAACTTCTCTGCATCATAACCTCTGCTTTCCTAATGGTTAC
m[12260]	SilicoDArT	Plus	C06	11,109,815–11,109,862	TGCAGATTTTGTGGCCGCCTTGGCGCGATCAAGATGCTTCTGATTAC
m[12402]	SNP	Minus	C06	19,867,263–19,867,332	TGCAGGAACGGTTGTGTGCAACGTTGATGCTGCGTGGAATGCCTCTTCTGGCCATTGTGGGCTTGGAGT
m[12405]	SNP	Plus	C06	19,907,824–19,907,893	TGCAGATCCACTATTTTTCCTATTCAAAGATCAGCCCTTTGTCCCTCTACCGCGGAGTTATATCCCTTC
m[12676]	SilicoDArT	Plus	C06	34,059,104–34,059,173	TGCAGTGCCTGAGATTGGTGATTGATAAAGCTTCTATATGAAACTTCTTTCTGACTCCAACTTTGGTGT
m[13394]	SilicoDArT	Minus	C07	40,184,137–4,0184,206	TGCAGCTTGCCTTCTTCTACATGGACTTCGATCTGGTATCCAAGAGCATTGACAAAGCTAAAAAGTAAG
m[13538]	SNP	Minus	C08_random	2,116,369–2,116,438	TGCAGAAGCTGTGAAGAAGCAAGAAGCTCTTGTCAAAGGGAAAGCGGTGGATAGTGAGAGGCACCAAGT
m[13703]	SilicoDArT	Plus	C08	12,855,108–12,855,177	TGCAGACTCCTCTAAACGAAGGAAGAAACCAAAATCTAAACACTCCAGCGGCGGATGTCTCCGCGGCCA
m[14408]	SilicoDArT	Minus	C09	23,726,449–23,726,518	TGCAGTATTACGATCCTCCGATGATCATATTGACTCTGTGGCGACAATTGTCGTTGCCCTTTGTTGTGG

* SNPs within the marker sequences underlined.

**Table 2:** Candidate genes linked to the markers identified at *p* < 0.01.

Marker	Candidate Genes
m[164]	Sequence localized within *BnaA01g05820D* (from 2nd intron to 3rd exon)
m[692]	Sequence localized within *BnaA02g02320D* (from 3rd exon to 3rd intron)
m[706]	Sequence localized within 2nd (last) exon of *BnaA02g03270D*
m[711]	Sequence localized within 4th (last) exon of *BnaA02g03340D*
m[712]	SNP localized within 10th exon of *BnaA02g03350D*
m[715]	SNP localized within 9th intron of *BnaA02g03360D*
m[717]	Sequence localized within *BnaA02g03360D* (from 9th exon to 9th intron)
m[2316]	Sequence localized between *BnaA04g03650D* (8101 bp from START codon) and *BnaA04g03660D* (1197 bp from STOP codon)
m[2318]	Sequence localized between *BnaA04g03710D* (2564 bp from START codon) and *BnaA04g03720D* (1917 bp from STOP codon)
m[2544]	SNP localized within 1st exon of *BnaA04g10630D*
m[3930]	Sequence localized within 2nd intron of *BnaA05g31580D*
m[4542]	Sequence localized within 5′UTR of *BnaA06g35290D*
m[7642]	Sequence localized within 3rd intron of *BnaC01g09590D*
m[7750]	Sequence localized within 1st exon of *BnaC01g12810D*
m[8588]	Sequence localized within *BnaC02g01780D* (from 1st to 2nd intron)
m[8699]	SNP localized within 5th exon of *BnaC02g07010D*
m[8700]	SNP localized within 5th intron of *BnaC02g07010D*
m[9871]	Sequence localized between *BnaC03g42220D* (12635 bp from START codon) and *BnaC03g42230D* (16337 bp from START codon)
m[11004]	Sequence localized within *BnaC04g27510D* (from 6th exon to 6th intron)
m[11154]	Sequence localized between *BnaC04g36110D* (15495 bp from START codon) and *BnaC04g36120D* (5975 bp from STOP codon)
m[11157]	Sequence localized between *BnaC04g36190D* (4515 bp from START codon) and *BnaC04g36200D* (5464 bp from STOP codon)
m[11160]	Sequence localized within 3′UTR of *BnaC04g36330D*
m[11177]	Sequence localized between *BnaC04g37750D* (7811 bp from START codon) and *BnaC04g37760D* (1064 bp from START codon)
m[11236]	Sequence localized within 5′UTR of *BnaC04g42180D*
m[12241]	Sequence localized between *BnaC06g09060D* (7068 bp from START codon) and *BnaC06g09070D* (30864 bp from STOP codon)
m[12260]	Sequence localized within 10th exon of *BnaC06g09300D*
m[12402]	SNP localized between *BnaC06g17150D* (4928 bp from START codon) and *BnaC06g17160D* (888 bp from STOP codon)
m[12405]	SNP localized between *BnaC06g17240D* (6459 bp from START codon) and *BnaC06g17250D* (11255 bp from START codon)
m[12676]	Sequence localized within 10th exon of *BnaC06g34710D*
m[13394]	Sequence localized within *BnaC07g39120D* (from 4th exon to 4th intron)
m[13538]	SNP localized between *BnaC08g47300D* (1373 bp from STOP codon) and *BnaC08g47310D* (22347 bp from START codon)
m[13703]	Sequence localized between *BnaC08g08600D* (51073 bp from START codon) and *BnaC08g08610D* (88405 bp from STOP codon)
m[14408]	Sequence localized between *BnaC09g25160D* (23907 bp from STOP codon) and *BnaC09g25170D* (3033 bp from STOP codon)

**Table 3:** Annotation of *Arabidopsis thaliana* orthologous genes. Data obtained from BnIR (https://yanglab.hzau.edu.cn/, accessed on 26 January 2026) and TAIR (https://www.arabidopsis.org/, accessed on 26 January 2026) databases.

*B. napus* Gene	*A. thaliana* Orthologue	*A. thaliana* Orthologue Protein Symbol (s)	Protein Description
*BnaA01g05820D*	*At4g31420*	REIL1	Cytosolic ribosomal 60S-biogenesis factor
*BnaA02g02320D*	*At5g14790*	-	ARM repeat superfamily protein
*BnaA02g03270D*	*At5g16820*	HSFA1b, HSF3	Transcription factor
*BnaA02g03350D*	*At5g17010*	VGT2	Major facilitator superfamily protein
*BnaA02g03360D*	*At5g17020*	XPO1A, XPO1, HIT2, ATCRM1	Member of the exportin protein family
*BnaA06g35290D*	*At5g47730*	SFH19	Sec14p-like phosphatidylinositol transfer family protein
*BnaC01g09590D*	*At4g28880*	CKL3	Protein serine/threonine kinase
*BnaC01g12810D*	*At4g21510*	FBS2	F-box family protein
*BnaC02g01780D*	*At5g06960*	OBF5, TGA5	Basic leucine zipper (B-ZIP) containing protein
*BnaC02g07010D*	*At5g17070*	PP4R2L	PP4R2 domain protein
*BnaC04g36330D*	*At3g25990*	GT-4	Homeodomain-like superfamily protein
*BnaC04g42180D*	*At2g31305*	INH3	A regulatory subunit of protein phosphatase 1 (PP1)
*BnaC06g09300D*	*At1g51980*	-	Insulinase (peptidase family M16) protein

## Data Availability

The data presented in this study are available upon request from the corresponding author.
